# Conjugation morphology of *Zygogonium ericetorum* (Zygnematophyceae, Charophyta) from a high alpine habitat

**DOI:** 10.1111/jpy.12363

**Published:** 2015-12-01

**Authors:** Rosalina Stancheva, Klaus Herburger, Robert G. Sheath, Andreas Holzinger

**Affiliations:** ^1^ Department of Biological SciencesCalifornia State University San MarcosSan MarcosCalifornia92096USA; ^2^ Institute of BotanyUniversity of InnsbruckSternwartestraße 15InnsbruckA‐6020Austria

**Keywords:** alpine habitat, conjugation, cytoplas‐mic residue, green algae, Zygnematophyceae, *Zygo‐gonium*

## Abstract

Reproductive characteristics are important for defining taxonomic groups of filamentous Zygnematophyceae, but they have not been fully observed in the genus *Zygogonium*. Specimens of *Z. ericetorum* previously studied and used to clarify the generic concept lacked fertile material, which was obtained recently. This study illustrates for the first time, using color light microscopic and fluorescence images, a consequent conjugation stage in *Z. ericetorum*, including completely developed zygospores and purple cytoplasmic residue content left outside the zygospores, similar to aplanospore formation. Structures confirmed earlier reports and provided new observation informative regarding phylogenetically relevant reproductive characters of *Z. ericetorum*.

**Abbreviations**
BP: band pass
LM: light microscopic
LP: long pass

Recently, a phylogenetic and morphological study of *Zygogonium ericetorum* Kützing, the type species of the genus, was conducted on a natural population from Austria, providing new data on the taxonomic importance of morphological characteristics of the genus (Stancheva et al. 2014). *Zygogonium* can be distinguished from other zygnematophytes by (i) irregular plate‐like chloroplasts; and (ii) purple cytoplasmic residual content left in sporangia outside of the fully developed aplanospores (Stancheva et al. 2014). Cytoplasmic residue outside of the zygospores was postulated, but could not be demonstrated as no zygospores were observed in the previous study. Such residues would imply similarities between asexual aplanospore and sexual zygospore formation. The main features of *Z. ericetorum* conjugation were described and illustrated by drawings (De Bary 1858, West and Starkey 1915, Hodgetts 1918, Transeau 1933, 1951, Kadlubowska 1984, Rundina 1998), but uncertainties exist. The only light microscopic (LM) documentation of conjugation in *Z. ericetorum* filaments (Alston 1958) did not allow one to draw conclusions, as neither color micrographs nor a description of the conjugation process were provided.

In additional sampling of the *Z. erictorum* population from the same location in Austria, we collected sexually reproducing filaments, and conducted LM and fluorescence microscopic observations of its conjugation morphology. Detailed LM observations and color images of conjugation in *Z. ericetorum* complete the understanding of its reproductive biology and the taxonomic significance of reproductive features. The implications of this knowledge can be used in determining the phylogenetic position and ancestral character state distribution within the wider group of closely related filamentous genera *Zygnema* and *Zygnemopsis*.

For this study, we obtained algal material from a natural population of *Z. ericetorum* growing in the same Austrian habitat in Mt. Schönwieskopf (46°50′998 N, 11°00′903 E), at 2350 m a.s.l. near Obergurgl, Tyrol, which has been previously sampled during the summer seasons 2007, 2008 (Holzinger et al. 2010), 2009, 2010, 2012 (Aigner et al. 2013), and 2013 (Stancheva et al. 2014). The chemical and physical conditions of the collection site have been described previously (Holzinger et al. 2010, Aigner et al. 2013); for this study, no new data of the collection site were recorded. We think that general climate data available for the collection site do not contribute to the understanding of the trigger for conjugation, an event that occurs only very locally at a specific‐habitat scale. *Z. ericetorum* samples were collected from several different areas in a spring pool on June 9, 2015 by the authors Stancheva and Holzinger, and kept cool until next day when they were processed in the laboratory in Innsbruck for light microscopy. Fresh conjugating filaments were observed and photographed with a Zeiss Axiovert 200M (Carl Zeiss AG, Jena, Germany) inverted fluorescence microscope equipped with a 63× Neofluor 1.4 NA objective lens. Images were captured with a Zeiss Axiocam MRc5 digital camera. To visualize chlorophyll autofluorescence, filaments were excited with a Zeiss filter set 09, excitation band pass (BP) 450–490, emission 515 nm long pass (LP). Blue autofluorescence of the cell walls was generated by Zeiss filter set 01 (excitation 365/12 nm, emission LP 397 nm).

The *Z. ericetorum* vegetative morphology was identical to the previous description by Stancheva et al. (2014). Cells of the filaments were 15–31 μm wide, containing two plate‐like green chloroplasts, thick multilayered cell walls, and frequent H‐shaped wall structures. Cell content was light purple. Several filaments formed zygospores or akinetes, but aplanospores were not observed.

The conjugation was scalariform, involving two, three or four filaments in irregular fashion (Fig. 1, A–E). Only a few cells in conjugating filaments took part in the reproduction (Fig. 1D), and abnormal gametangia with uncompleted conjugation were common (Fig. 1A). The conjugation process started with compaction of the chloroplasts, nucleus, and other cell organelles into the gamete, which migrated toward the conjugation tube formed between two corresponding cells in the adjacent filaments. The two gametes united directly in the tube and formed a zygospore separated by a gametangial wall from the purple cytoplasmic residue, consisting of a large vacuole remaining in the conjugating cells (Fig. 1, A, B, D, E). The gametangial wall became the wall of the zygospore. The zygospores were ovoid or ellipsoid, 15–26 μm wide, 19–38 μm long (Fig. 1, A, D, F–H) containing four compacted green chloroplasts, each with a single pyrenoid (Fig. 1, A and C), translucent cytoplasm, and smooth colorless or yellowish multilayered spore wall (Fig. 1, F–H). The zygospores did not develop a germination suture in their own wall (Fig. 1, F–H). The conjugation tubes were easily separated to discharge the zygospores due to distinct rupture along the contact line between them, which is visible as an equatorial line in the surface of the zygospores (Fig. 1, A and B). Zygospore germination was not observed.

**Figure 1:**
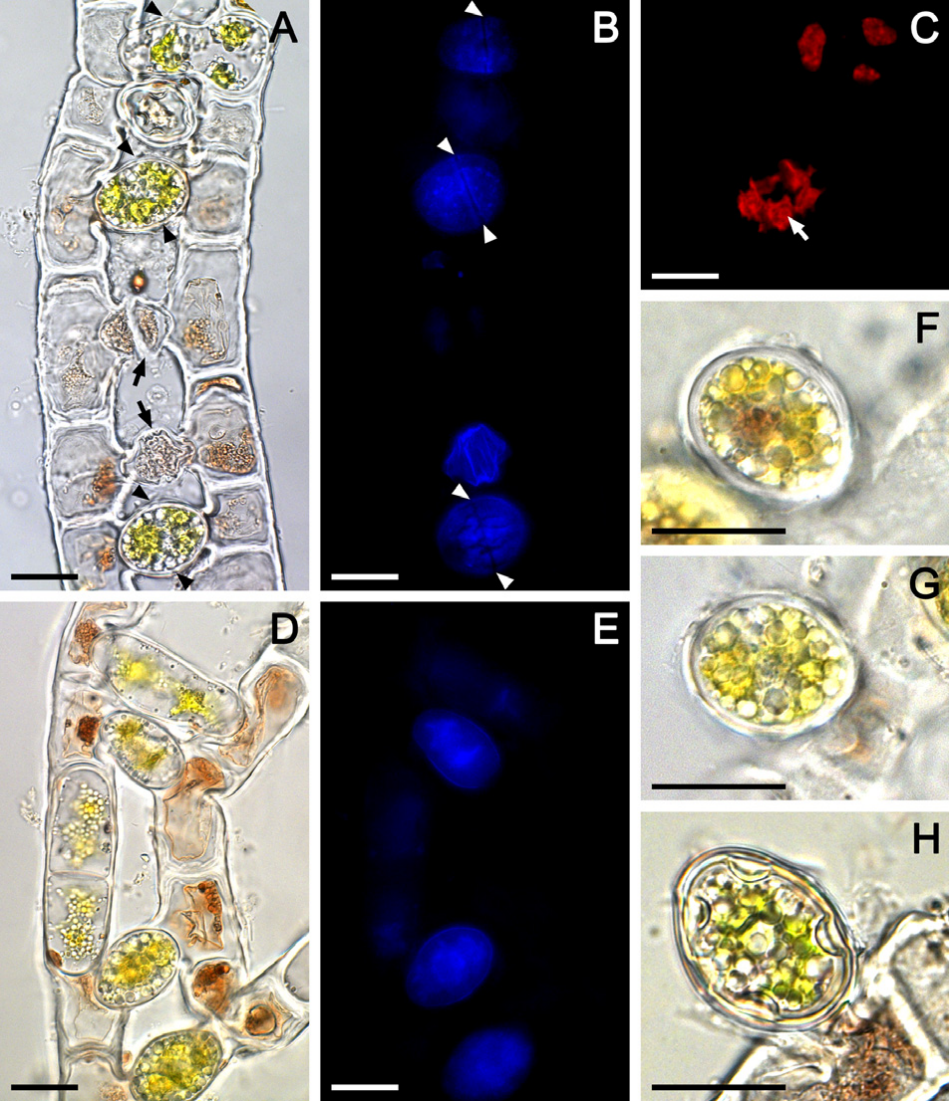
Light microscopic (A, D, F–H) and autofluorescence (B, C, E) images of consequent conjugation stages in *Zygogonium ericetorum*: (A–C) same conjugating filaments showing healthy united gametes and zygospores, and abnormal gametangia with incomplete conjugation (black arrows), (B) blue autofluorescence of zygospore cell wall compounds, arrowheads show the rupture along the contact line between the two corresponding conjugation tubes enclosing healthy united gametes and zygospores, (C) red autofluorescence of chloroplasts in gametes and zygospore, white arrow shows the pyrenoid, (D, E) same conjugating filaments in early conjugation stage, note that the united gametes and zygospores are separated from the purple‐colored cytoplasmic residue left in gametangia by wall, (F, G) zygospores with thick smooth multilayered colorless spore wall detached from one of the conjugating filament, (H) completely developed zygospore with thick smooth multilayered yellowish spore wall detached from one of the conjugating filament; scale bars: 20 μm.

Taxonomically, reproductive characteristics are very important for defining groups of filamentous Zygnematophyceae, but they are infrequently observed in natural populations. The scarcity of conjugation in *Z. ericetorum* combined with common abnormalities (Fig. 1A) due to its strong tendency toward encystment, whenever habitat conditions change (Transeau 1951), contributed to misinterpretations of this process in the early studies (De Bary 1858, Hodgetts 1918). This study confirmed the postulation that characteristics of the aplanospore formation described in our previous work (Stancheva et al. 2014) are applicable to the zygospore formation, which is generally valid for the whole family Zygnemataceae (Transeau 1951). We demonstrated that during the conjugation process in *Z. ericetorum*, similar to the aplanospore formation, the colored cytoplasmic vacuolar residue is left outside the zygospore. Thus, the spore lacks purple pigmentation, but contains cell organelles and storage products (Fig. 1, F–H). Excretion of the purple pigment during both zygospore and aplanospore formation might be a protective strategy to avoid toxic pigment concentrations in the newly formed spore.

Furthermore, the color LM and fluorescent microscopic images of fertile material revealed that the zygospore wall is smooth, multilayered, colorless to yellowish. The previous descriptions of this species typically omit the color of the zygospore wall (e.g., Transeau 1933, 1951, Alston 1958), or it was reported as colorless or yellow‐brown (Kadlubowska 1984), yellow to brown (Rundina 1998), or dark brown (Johnson 2011). It is possible that in *Z. ericetorum,* the color of the zygospore wall is variable, but our observations showed that the aplanospores and zygospores are most likely lacking special spore‐wall pigment and they are typically colorless (Stancheva et al. 2014, this study). This observation is in contrast to the closely related genus *Zygnema*, which was phylogenetically divided into two main groups based on the blue versus brown color of the mesospore wall layer (Stancheva et al. 2012), indicating the importance of the spore wall color as an evolutionary character in Zygnemataceae. The genus *Zygnema* includes many species that form zygospores in the conjugation tubes; some of these species appear similar to *Zygogonium* in their mode of gamete fusion, as well as zygospore and gametangial wall formation. Since the formation of zygospores in tubes versus in gametangia in *Zygnema* is phylogenetically informative (Stancheva et al. 2012), a very interesting question for further studies is which *Zygnema* group is most closely related to *Zygogonium*. For instance, *Zygnema sterile* Transeau is the only species that produces akinetes with colorless walls, similar to *Z. ericetorum*, but its conjugation is unknown, and it is phylogenetically close to species with blue mesospores (Stancheva et al. 2012).

Some authors (Transeau 1951, Yamagishi 1963, Johnson 2011) considered that the presence of a special gametangial wall in *Zygogonium* distinguished it from *Zygnema*, but our observations in both genera did not support this view (compare fig. 2, Q and R in Stancheva et al. 2012, and Fig. 1, A and D in this study). Transeau (1951) stated that there is an equatorial suture in the gametangial wall in *Zygogonium*, but this study showed that the equatorial rupture is actually within the superficial layer of the conjugating tubes above the spore wall (Fig. 1, A and B). In addition, the work revealed new specifications of the zygospores determining the germination mode and beeing relevant to for the generic concepts of *Zygogonium* and *Zygnema*, which were not noticed before, e. g., the lack of germination suture in the zygospore wall (Fig. 1, F–H), similarly to aplanospores (Stancheva et al. 2014). In *Z. ericetorum*, the spores germinated through cell enlargement and spore cell division as documented for aplanospores (see figs. 4C and 5, C, D in Stancheva et al. 2014). We did not observe zygospore germination, but a similar germination mode was illustrated for zygospores by Hodgetts (1918) (Fig. 2F). By contrast, in *Zygnema,* the spore germinates along the germination suture in the mesospore, and the most inner endospore wall layer becomes the cell wall of the new filament (see fig. 2O in Stancheva et al. 2012).

In conclusion, this study provided new data on reproductive morphology of *Z. ericetorum*, which improves the understanding of its conjugation process and phylogenetically relevant reproductive characters.

Traveling of A.H. was supported by a grant from the University of Innsbruck, International Relations Office. The study was supported with funding from the California State Water Resources Control Board Consolidated Grants and SWAMP Programs to R. G. S. and R. S., and by the Austrian Science Fund (FWF) grants P 24242‐B16 and I 1951‐B16 to A. H.
